# Influence of the Training Process on the Health Literacy of Angolan Health Promoters

**DOI:** 10.3390/ijerph22091358

**Published:** 2025-08-29

**Authors:** Manuela Ferreira, Eduardo Santos, Joana Andrade, Inês Figueiredo, Vitor Martins, Sofia Campos

**Affiliations:** 1 Health Sciences Research Unit: Nursing (UICISA: E), Nursing School of Coimbra (ESEnfC), Health School of the Polytechnic Institute of Viseu, 3504-510 Viseu, Portugal; ejf.santos87@gmail.com (E.S.); sofiamargaridacampos@gmail.com (S.C.); 2 Unidade Local de Saúde de Viseu Dão-Lafões, E.P.E, 3504-509 Viseu, Portugal; joanaandrade@saudeportugues.org (J.A.); inesfigueiredo@saudeportugues.org (I.F.); vitormartins@saudeportugues.org (V.M.)

**Keywords:** health literacy, health promoters, Gungo, Angola

## Abstract

**Background:** As part of the research project ‘Seigungo–Gungo’s Health, Education, and Maternal and Child Quality of Life: An Action-Research Project’, a study was conducted in the Gungo community in Angola, a region facing significant challenges in terms of access to healthcare and health literacy. The primary aim of the study was to evaluate the effectiveness of an intervention training model designed to improve the health literacy of the participants. **Methods**: The sample consisted of 30 trainees, 60% of whom were male, with an average age of 45.6 years. Most participants were single (53.3%) and had completed 6 years of formal education (26.7%). Health literacy levels were assessed using the HLS-EU-PT-Q16, a short 16-item questionnaire designed to assess three key domains: healthcare, disease prevention, and health promotion. These domains are related to the focus of the training programme. Data collection took place throughout the year 2024. **Results**: According to the data obtained, prior to attending the training program, 60% of the participants demonstrated an inadequate level of health literacy. Following the intervention, this percentage dropped significantly to 20%. In contrast, the proportion of participants with sufficient to excellent health literacy rose from 16.7% to approximately 40%. The results indicate that the training program had a positive and statistically significant impact on improving health literacy in the Gungo community. **Conclusions**: These findings highlight the importance of targeted training and sustained intervention efforts to address the specific health education needs currently affecting this community.

## 1. Introduction

According to data from UNESCO, in 2022, the literacy rate in Angola for people aged 15 years and above was 72.4% [1]. Several international organisations have raised concerns about the state of the right to education and health for all Angolans. According to the World Health Organisation (WHO), the country has an average medical coverage of 244 doctors per 10,000 inhabitants [2], which is significantly below the standard average among the Organisation for Economic Co-operation and Development (OECD) countries, which is around 3.3 doctors per 1000 inhabitants [3].

Gungo is a low-resource town located in the Kwanza Sul province and forms part of the municipality of Sumbe, Angola. In 2016, researchers from the Seigungo project performed a diagnostic screening, and only 10 health centres were identified in the commune, all of which face severe shortages of both human and material resources. Challenges are further aggravated by the rural exodus of qualified professionals and individuals with higher academic qualifications. On the other hand, cultural issues, often rooted in mysticism and beliefs associated with witchcraft, frequently influence health-related decision-making. The lack of basic hygiene conditions further increases health risks for patients.

In addition to the region’s widespread poverty, levels of health literacy remain critically low. Definitions of health literacy have significantly evolved over time, influenced by advances in science and technology, changes in healthcare provision, and growing expectations stemming from new perspectives on individual, group, and societal responsibility [4,5,6,7]. While international consensus on a precise definition of health literacy has yet to be reached, there is a strong commonality between the various definitions and models studied. Despite their complexity and heterogeneity, these definitions consistently describe health literacy as a multidimensional and intricate construct [8]. In recent years, health literacy has been widely recognized as an essential competency for promoting health, reducing inequalities, and empowering individuals and communities. The concept has evolved significantly, encompassing not only the ability to read and understand written health information but also the ability to access, critically evaluate, and use this information effectively in everyday health-related decision-making [9]. More recent literature has reinforced the dynamic and contextual nature of health literacy, emphasizing that it should be understood as a social construct interconnected with structural determinants such as poverty, education, gender, and equity in access to care [10]. According to Sørensen et al. [11], strengthening health literacy is crucial to achieving the Sustainable Development Goals (SDGs), particularly in highly vulnerable contexts, such as the Gungo commune in Angola, where there is a critical intersection between low levels of education, extreme poverty, and limited access to basic services. Thus, promoting health literacy, especially among community health workers, proves to be a central strategy for mitigating cultural and structural barriers and fostering effective self-care and prevention practices adapted to the local context.

According to the WHO [12], the promotion of health literacy not only benefits individuals but also extends to the wider community and aligns with several of the United Nations’ Sustainable Development Goals outlined in the 2030 Agenda, namely SDG 1—No Poverty, SDG 2—Zero Hunger, SDG 4—Quality Education, and SDG 10—Reduced Inequalities, among others mentioned.

In this context, health literacy plays a crucial role in ensuring that populations have access to the information they need to make informed decisions about their health. In the case of the Gungo commune, several factors influence the population’s health literacy levels. These include language barriers, limited access to health services, and pronounced socio-economic inequalities. Together, these challenges hinder individuals’ ability to obtain, process, and understand basic health information, ultimately limiting their capacity to make informed decisions about their health and adopt preventive behaviours.

Since health literacy is a key factor in changing risk behaviours and enhancing existing health practices, the primary aim is to evaluate the effectiveness of an intervention training model designed to improve health promoters’ literacy in the Gungo community.

## 2. Materials and Methods

### 2.1. Study Design

This is a quantitative, observational, before-and-after study using a descriptive-correlational analysis that was conducted in accordance with the STrengthening the Reporting of OBservational studies in Epidemiology statement guidelines (STROBE) [13]. SEiGungo is an action research project conducted in the Gungo community (Kwanza Sul, Angola). Berku [14] suggests that action research should extend beyond the study of past actions and include a critical analysis of current procedures and the development of strategies for their improvement. This approach combines practice with critical reflection to address specific problems within the educational context in which it is developed. The SEiGungo project aimed to foster human development by providing sustained and continuous training for the human resources responsible for delivering quality maternal and child healthcare.

The project also sought to test an intervention training model designed to promote positive changes in the social and economic determinants of health. To this end, and following a diagnostic assessment, 30 participants (25 health promoters and midwives and 5 nurses from the Kwanza Sul Polytechnic Institute) participated as trainees in approximately 360 h of theoretical and practical instruction, organized into one-week modular sessions. The training program developed was carefully designed and adapted to the vulnerable context of Gungo. The training program was designed to address the gaps identified in areas such as preconception care, family planning, pregnancy, childbirth, puerperal care, neonatal and paediatric care, and health literacy. The training program’s topics included fundamental principles in health and education, sexual and reproductive health, and neonatal, child, and paediatric care. The intervention team consisted of nurses, doctors, and a psychologist from Portugal. Active pedagogical methodologies were used throughout the course, including problem-solving strategies, master learning or reflection, problematization of reality, and debates. These teaching-learning methods not only aim to teach content but also to develop the personal and interpersonal skills necessary for the work of health promoters in Gungo. Strategies were implemented to promote engagement, critical thinking, and reflective thinking, giving trainees the opportunity to obtain immediate feedback. Video, image, and simulated practice resources were also used. Furthermore, during the training, professors from the health area of the South Kwanza Polytechnic Institute were present, who acted as facilitators to bridge the gap between the Portuguese trainers and the Angolan health promoters, helping in the understanding of the language used during the training process. This program was divided into two stages: an initial screening in October 2023, followed by theoretical and practical instruction in January 2024, and post-training data collection in October 2024. The study employed a non-probability convenience sampling method. Data were collected using two instruments: a sociodemographic questionnaire developed to characterize the sample and the HLS-EU-PT-Q16 questionnaire [15]. The HLS-EU-PT-Q16 is a shortened version of the European Health Literacy Survey Questionnaire, composed of 16 items that cover three key domains: healthcare, disease prevention, and health promotion. These 16 items were dichotomized: responses marked as ‘difficult’ and ‘very difficult’ were assigned a value of 0, while ‘easy’ and ‘very easy’ answers were assigned a value of 1. The total score, obtained by summing the values of all 16 items, reflects each participant’s level of health literacy. A score of 13 or higher indicates an “adequate” health literacy, scores between 9 and 12 indicate a “problematic” health literacy, and scores of 8 or below indicate an “inadequate” health literacy. To ensure effective comparison between subdomains, scores were standardized using the General Health Literacy Index (GHLI), based on a variable metric scale ranging from 0 to 50. The G-HL16 index was calculated as follows: G-HL16 index = (mean − 1) × (50/3). Four HL levels were defined: inadequate (0–25), problematic (25.1–33), sufficient (33.1–42), and excellent (42.1–50). The questionnaire demonstrated strong overall internal consistency (α = 0.89). Internal consistency within the “Health care” (α = 0.78), “Disease prevention” (α = 0.72), and “Health promotion” (α = 0.70) subdomains was also acceptable. The final validation of the instrument is being carried out and will be published in a specialized journal.

Data collection for this study began before formal approval was granted by the Ethics Committee of the Instituto Politécnico de Viseu on 24 April 2025 (Reference Nº19/SUB/2025), because there was no ethics committee in the Gungo region, and it was necessary to comply with the training calendar stipulated by the Portuguese trainers. However, all ethical safeguards were ensured, including written informed consent, culturally adapted oral explanations, and privacy protection. No procedure that could be considered ethically unacceptable was performed. Data collection was carried out using a written questionnaire on paper, administered by a researcher assigned to the field, ensuring the confidentiality and anonymity of the participants’ responses, as well as the voluntary nature of their participation. After the compilation of the database, the data were stored online and protected by a password accessible only to the members of the research team. It was retained solely for the period necessary to achieve the purposes for which it was collected and will be subsequently deleted in its entirety. Statistical analysis was conducted using IBM^®^ SPSS^®^ Statistics software, version 29.0. A descriptive analysis of the data were performed to calculate absolute frequencies (n), percentages (%), measures of central tendency (mean—M), and the measure of dispersion (standard deviation—SD). The reliability of the instrument was assessed using Cronbach’s alpha (α) and McDonald’s omega (ω) coefficients. The following threshold values were established: >0.9 (excellent); between 0.8 and 0.9 (good); between 0.7 and 0.8 (acceptable); between 0.6 and 0.7 (questionable); between 0.5 and 0.6 (poor); and <0.5 (unacceptable).

Prior to any analysis, the assumptions of normality were confirmed. The skewness and kurtosis values, as well as the value of the Shapiro–Wilk test statistic and its *p*-value, indicated that the data did not follow a normal distribution (*p* < 0.05). In this sense, non-parametric tests, namely the Wilcoxon test, were used for inferential analysis to compare the means of a quantitative variable in paired groups across two observation points. A value of *p* < 0.05 was considered statistically significant.

### 2.2. Sample Characterization

The sample consisted of 30 participants, the majority of whom were male (60%) with a mean age of 45.57 ± 10.86 years (range 24–64 years). Ten to fifteen participants are frequently used in participatory action research. Larger samples are usually used for quantitative characteristics in order to guarantee statistical significance [16]. Nevertheless, we calculated the sample size using G*Power 3.1.9.7, assuming an effect size of 1.05 based on the mean and standard deviation parameters of the groups, an alpha of 0.05, and a power of 0.95, requiring a minimum sample of 15 participants. Most participants were single and had completed six years of formal education. The majority reported being displaced from their usual place of residence and stated they were living with other family members. Participants had an average of 13.82 ± 8.47 years of work experience (range 1–36 years) (Table 1).

Regarding lifestyle, most of the participants reported never having smoked and indicated they had not consumed alcoholic beverages in the past year. The majority indicated they did not use a computer/internet daily (Table 2).

## 3. Results

Table 3 shows the responses to the HLS-EU-PT-Q16 questionnaire that were collected during the diagnostic assessment conducted prior to the participants ‘enrolment in the training program. Items 1, 4, 10, and 15 recorded the highest percentage of “very difficult” responses (23.2%,13.3%, 13.3%, and 13.3%, respectively). In contrast, the highest percentages of “very easy” responses were observed in items 1, 2, and 8 (13.3%, 20%, and 13.3%, respectively) (Table 3).

Cronbach’s alpha and McDonald’s omega coefficients for the internal consistency of the HLS-EU-PT-Q16 were, respectively, as follows: 0.87 and 0.86 for the healthcare subdomain, 0.81 and 0.81 for the disease prevention subdomain, 0.72 and 0.72 for the health promotion subdomain, and 0.91 and 0.91 for the overall HLS-EU-PT-Q16 scale. Considering the different calculation methods, it should be noted that prior to attending the training program, 83.3% of the participants revealed inadequate or problematic health literacy levels according to the HL index. A similar trend was observed when the sum of the individual item scores was used: in this particular case, 86.7% of participants also exhibited inadequate or problematic health literacy levels. After completing the training program, a significant increase was observed in the proportion of participants attaining sufficient to excellent health literacy. According to the HL index, the percentage rose from 16.7% to 40% (*p* < 0.001) while the item score method indicated an increase from 13.3% to 66.7% (*p* = 0.006) (Table 4).

## 4. Discussion

The primary aim of the study was to evaluate the effectiveness of a training intervention model designed to improve participants’ health literacy. Based on the data presented, this objective was successfully achieved. The data show that, prior to attending the training programme, 60% of the participants had an inadequate level of health literacy. Following the intervention, this scenario decreased to 20%. Conversely, the proportion of participants with sufficient to excellent health literacy levels increased from 16.7% to approximately 40%. This significant improvement in health literacy levels suggests that the training program effectively contributed to increasing knowledge acquisition, which may, in turn, play a crucial role in fostering attitude changes and promoting healthier choices. Furthermore, throughout its different stages, the project was adapted to the local cultural environment, and strategies and dynamics designed to engage both the communities and the trainees were implemented. The use of culturally sensitive methods and participatory pedagogic actions proved to be critical for the trainees’ motivation and involvement and helped to better transmit health-related messages. In low-resource locations, other previous literature has found similar findings [17,18]. These efforts facilitated the transmission of knowledge and were key in demystifying culturally rooted beliefs, supporting decision-making in crisis situations, and developing critical thinking about health-related decisions among the population.

The trainees came from a region marked by poor infrastructure and a shortage of health professionals, which clearly limits the population’s access to adequate information about health promotion and disease prevention.

Although knowledge alone may not be enough to alter behaviours, it provides a critical foundation for triggering attitudinal changes that can contribute to more informed and appropriate health-related decisions [19].

Personal knowledge and competencies are mediated by organisational structures and the availability of resources that enable individuals to access, understand, appraise, and use information and services to promote and maintain good health and well-being for themselves and those around them [12]. In addition, ref. [20] highlights the importance of interaction and participation within the community and broader society as key components of health literacy [8,20].

Lower levels of health literacy are associated with a reduced capacity to make rational and informed decisions, which can often result in more reactive and less effective health-related choices [21]. In our study, 83.3% of the trainees- local health promoters- were found to have inadequate or problematic levels of health literacy according to the HL index.

This study has some limitations. The sample could have been larger, even though it met the minimum requirement and allowed statistically significant differences to be found. The major limitation is that the HLS-EU-PT-Q16 instrument has not yet been formally validated. As mentioned above, validation is currently underway and will be published in another specialised publication dedicated to the study of its psychometric properties. Nevertheless, it should be noted that a cultural validation process was carried out, and the researchers ensured that the instrument was well understood before its application, which, in a way, guarantees the preliminary face validity of the instrument. The study of internal consistency, using Cronbach’s alpha and McDonald’s omega, also showed acceptable to good levels in the domains of the instrument and in the overall score, respectively. Finally, it should be noted that the results of Santos et al. [22] already pointed to the possibility of using the HLS-EU-PT^®^ instrument in the Angolan context, which motivated us to use it. Although it is not the Q16 version that we used, this version already attests to the feasibility of using and validating the HLS-EU-PT^®^ instrument.

## 5. Conclusions

The objectives we set by implementing the training program for health promoters were generally achieved. The results demonstrate that the training program had a positive and statistically significant impact on enhancing health literacy in the Gungo community, particularly in the areas of health care, disease prevention, and health promotion. These findings further support the need for ongoing training and health interventions in this community, which leads us to conclude that the training program was effective and met its proposed objectives.

The lack of accessibility to health care and education demonstrates a direct impact on health literacy, as it limits individual capacity to access, understand, and effectively use health information. Restricted access to formal education can hinder the ability to interpret medical information, understand diagnoses, and adhere to health recommendations. In the population studied, poverty further limits access to essential health services, medications, and diagnostic tests, making it even more difficult for individuals to make informed health decisions.

Overcoming these challenges requires the implementation of effective public policies, sustained investments in education and healthcare, and the development of strategies to ensure that information is accessible to all. Educational campaigns, easier access to health services, and targeted training programmes can play a crucial role in empowering individuals to take better care of their own health. Promoting health literacy among these highly vulnerable populations can help reduce inequalities and improve health outcomes. Health professionals should, through effective health education, encourage attitudes that support healthy lifestyles and foster the development of competencies that are essential for adopting and maintaining positive health behaviours. Such interventions are particularly important in communities like Gungo, where social and economic barriers significantly hinder access to health knowledge and services.

## Figures and Tables

**Table 1:** Sociodemographic background.

Variables	*n*		%	
Gender				
Male	18		60	
Female	12		40	
Marital status				
Single	16		53.3	
Married or Cohabiting couples	14		46.7	
Level of education				
3 years	1		3.3	
4 years	2		6.7	
6 years	8		26.7	
8 years	4		13.3	
9 years	4		13.3	
11 years	2		6.7	
12 years	2		6.7	
13 years	2		6.7	
Bachelor’s degree	5		16.7	
Displaced from usual residence				
Yes	24		80	
No	6		20	
Household				
Living alone	2		6.7	
Family of origin	6		20	
Other family members	21		70	
Other	1		3.3	
	M	Dp	Min	Max
Age	45.57	10.86	24	64
Years of work experience	13.82	8.47	1	36

M: mean; DP: standard deviation; Min: minimum; Max: maximum.

**Table 2:** Lifestyle characterization.

Variables	*n*	%
Smoking		
Used to smoke, but quit	1	3.3
Never smoked	29	96.7
Current smoker		
No	30	100
Alcohol consumption in the past year		
Yes	3	10
No	27	90
Consumption of five or more alcoholic drinks in a single day during the past year		
Once a month	3	10
Never	26	86.7
Don’t know/no answer	1	3.3
Alcohol consumption in the past month		
Yes	3	10
No	26	86.7
Don’t know/no answer	1	3.3
Daily frequency of computer/internet access		
I don’t use it daily	21	70
Less than 1 h	7	23.3
Between 1 and 4 h	2	6.7

**Table 3:** Responses to the HLS-EU-PT-Q16 (*n*; percentages).

Domain	On a Scale from Very Easy to Very Difficult, How Easy Would You Say It Is for You to	Don’t Know/No Answer	Very Easy	Easy	Difficult	Very Difficult
HC	1. Find information on treatments of illnesses that concern you	2; 6.7%	4; 13.3%	6; 20%	11; 36.7%	7; 23.3%
HC	2. Find out where to get professional help when you are ill?	1; 3.3%	6; 20%	5; 16.7%	17; 56.7%	1; 3.3%
HC	3. Understand what your doctor says to you?	1; 3.3%	1; 3.3%	4; 13.3%	21; 70%	3; 10%
HC	4. Understand your doctor’s or pharmacist’s instructions on how to take a prescribed medicine	-	3; 10%	4; 13.3%	19; 63.3%	4; 13.3%
HC	5. Judge when you may need to get a second opinion from another doctor?	1; 3.3%	2; 6.7%	12; 40%	13; 43.3%	2; 6.7%
HC	6. Use information the doctor gives you to make decisions about your illness?	2; 6.7%	-	11; 36.7%	15; 50%	2; 6.7%
HC	7. Follow instructions from your doctor or pharmacist?	-	1; 3.3%	6; 20%	20; 66.7%	3; 10%
DP	8. Find information on how to manage mental health problems like stress or depression.	4; 13.3%	4; 13.3%	16; 53.3%	4; 13.3%	2; 6.7%
DP	9. Understand health warnings about behaviour such as smoking, low physical activity, and drinking too much.	5; 16.7%	2; 6.7%	7; 23.3%	13; 43.3%	3; 10%
DP	10. Understand why you need health screenings.	9; 30%	3; 10%	11; 36.7%	3; 10%	4; 13.3%
DP	11. Judge if the information on health risks in the media is reliable?	6; 20	1; 3.3%	16; 53.3%	5; 16.7%	2; 6.7%
DP	12. Decide how you can protect yourself from illness based on information in the media.	3; 10%	1; 3.3%	16; 53.3%	9; 30%	1; 3.3%
HP	13. Find out about activities that are good for your mental well-being.	5; 16.7%	2; 6.7%	9; 30%	11; 36.7%	3; 10%
HP	14. Understand advice on health from family members or friends?	1; 3.3%	3; 10%	6; 20%	17; 56.7%	3; 10%
HP	15. Understand information in the media on how to get healthier?	3; 10%	3; 10%	5; 16.7%	15; 50%	4; 13.3%
HP	16. Judge which everyday behaviour is related to your health?	6; 20%	2; 6.7%	10; 33.3%	11; 36.7%	1; 3.3%

HC: health care; DP: disease prevention; HP: health promotion.

**Table 4:** Level of health literacy.

	Prior to Attending the Training Program		After Completing the Training Program		*p*
	*n*	%	*n*	%	
Level of health literacy (HL index)					
Inadequate	18	60	6	20	
Problematic	7	23.3	12	40	
Sufficient	5	16.7	11	36.7	
Excellent	0	0	1	3.3	
Total score	M = 8.3; SD = 3.76		M = 12.53; SD = 4.25		<0.001 *
Level of health literacy					
Inadequate	14	46.7	5	16.7	
Problematic	12	40	5	16.7	
Adequate	4	13.3	20	66.7	
Total score	M = 22.32; SD = 9.57		M = 29.58; SD = 9.26		0.006 *

* Statistically significant; M: mean; SD: standard deviation.

## Data Availability

Data are available upon reasonable request.
